# Participation in physical activity decreased more in people with rheumatoid arthritis than the general population during the COVID-19 lockdown: a cross-sectional study

**DOI:** 10.1007/s00296-021-05054-4

**Published:** 2021-11-30

**Authors:** Christopher Balchin, Ai Lyn Tan, Oliver J. Wilson, Jim McKenna, Antonios Stavropoulos-Kalinoglou

**Affiliations:** 1 Leeds Beckett University, Carnegie School of Sport, Leeds, UK; 2 University of Leeds, Leeds Institute of Rheumatic and Musculoskeletal Medicine, Chapel Allerton Hospital, Leeds, UK; 3 Leeds Teaching Hospitals NHS Trust, NIHR Leeds Biomedical Research Centre, Leeds, UK

**Keywords:** Rheumatoid arthritis, COVID-19, Physical activity, Mental wellbeing

## Abstract

The COVID-19 pandemic and social distancing restrictions have significantly reduced population-wide physical activity (PA) levels. However, the impact of the pandemic and relevant restrictions on PA participation, and any potential barriers to it, in people with rheumatoid arthritis (RA) are not clear. Furthermore, we are unsure if any such PA changes have affected their body weight, mental wellbeing, and/or quality of life (QoL). Thus, the aim of this study was to examine the impact of the lockdown on PA participation in people with RA, versus people without RA. Participants (*n* = 128; RA = 27, non-RA = 101) completed a self-administered online survey, which included questions on PA, body weight, mental wellbeing and QoL. PA participation during lockdown was significantly lower among RA versus non-RA participants (*p* < 0.001). Additionally, a similar profile of results was found where more RA participants vs non-RA participants reported reduced habitual PA (59% vs 33%) and increased body weight (59% vs 35%). Mental wellbeing scores were similarly low in both groups during lockdown (RA: 20.8 ± 4.2; non-RA: 22.2 ± 3.4, *p* = 0.080). Matched group comparisons identified similar trends to full sample analyses. In the first months of the lockdown, more people with RA reported decreased PA participation and increased body weight than their non-RA counterparts. Access to exercise equipment and facilities appears to be the main cause for these results. Looking beyond COVID-19, specific PA promotion for people with RA will be required to prevent a pandemic of inactivity.

**Supplementary Information:**

The online version contains supplementary material available at 10.1007/s00296-021-05054-4.

## Introduction

Rheumatoid arthritis (RA) is the most common chronic inflammatory arthritis [1]; typical symptoms include joint pain and stiffness limiting mobility [2]. Physical inactivity and sedentary behaviour are highly prevalent in people with RA [3]. Fear for exacerbating disease symptoms (i.e. pain) and misconceptions regarding safety are commonly cited barriers to physical activity (PA) in this population [4]. Importantly, levels of PA in RA associate with mental wellbeing, quality of life (QoL) [5] and disease severity [6].

The emergence of the novel coronavirus (SARS-CoV2) and its associated disease (coronavirus disease 19; COVID-19) forced many countries to enforce restrictions on citizens mobility, in an attempt to contain the spread of the virus. In the UK, this included a stay-at-home order, closure of non-essential businesses (e.g. gymnasiums, restaurants and retail) and self-isolation for any individuals presenting with COVID-19 symptoms [7]. People were allowed to exit their homes for specific reasons, such as essential shopping, doctor’s appointment, travel to work, or one form of exercise per day [7]. However, a part of the population was advised to follow even more stringent measures of self-isolation. Shielding, as referred to by Public Health England [8] applied to many people with chronic conditions such as diabetes, cancer and RA [3]. These people were advised to remain at home, not come in contact with anyone else, even other members of the same household [8].

These lockdown measures seem to have significantly impacted on the population-wide levels of PA; in the UK step counts have fallen by ~ 30% following introduction of the lockdown and remained well below pre-lockdown levels until June 2020 [9]. This sustained period of reduced PA may have adverse health implications [10]. Indeed, among people with RA, PA is a major determinant of obesity [11] and even associates with disease activity and frequency of hospitalisation [6]. Therefore, it is important to understand how the pandemic may have impacted PA and associated health aspects of people with RA. The aim of this study was to evaluate the impact of lockdown on PA participation and any barriers to it, type of exercise performed, body weight changes, mental wellbeing and QoL in people with RA versus people without RA.

## Methods

### Participants

A convenience sample approach was used where a total of 165 participants were recruited using study adverts shared via online advertisements and invitations to participate uploaded on social media platforms (i.e. Facebook, Instagram and Twitter). However, as lockdown rules varied across countries, only data from 128 UK residents was included (RA = 27, non-RA = 101) and 37 non-UK residents were subsequently excluded. Inclusion criteria for people with RA was self-reported clinical diagnosis. All participants were aged over 18 years old and provided informed consent for their data to be used for research purposes before completing a self-administered online open survey (Qualtrics XM, United States). Ethical approval was granted by Leeds Beckett University Ethics Committee (application ID: 71085). No incentives were offered to participants and participation was entirely voluntary.

### Survey design

The survey was only available in English, using clear, unambiguous and well-articulated language [12] in order to minimize response bias. Close-ended questions were the main component of the survey while open-ended questions were used minimally. Furthermore, to reduce the range of responses for certain questions slider scales were used [13, 14], as well as check boxes in questions with multiple answers. Also, matrix scales were used to combine multiple questions with a similar range of answers (see Online Resource 1 for specific questions). The participant information sheet included the purpose of the survey, the length of time to complete the survey and the contact details for those conducting it. There was also a clear description of the data anonymization process and how personal information was stored. As part of survey validation, pilot testing was performed by the investigating team to identify and correct deficiencies in the survey design, while functionality and logic was manually checked. Additionally, test–retest reliability was completed during survey validation, which confirmed the consistency of questions.

**Fig. 1:**
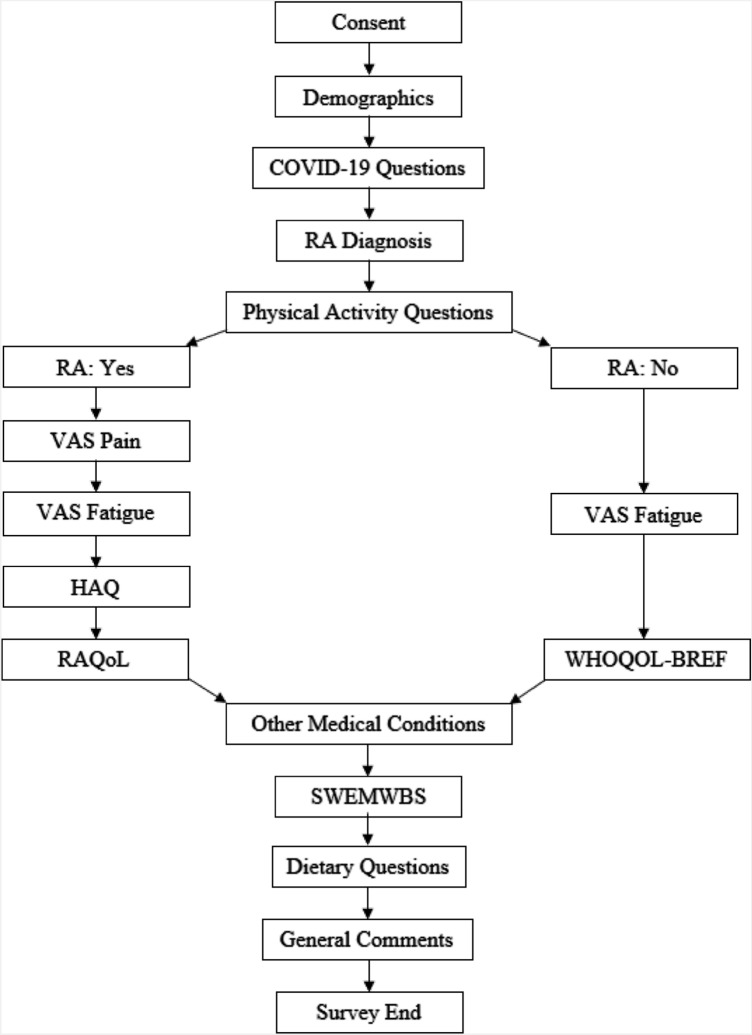
Flow diagram of survey design. The arrows indicate the order in which the survey was completed. Those who responded yes to RA diagnosis, answered questions relating to disease characteristics (i.e. VAS pain, VAS fatigue, HAQ and RAQoL) while those who responded no to RA diagnosis, completed VAS fatigue and the WHOQOL-BREF. RA, rheumatoid arthritis; VAS pain, visual analogue scale pain; VAS fatigue, visual analogue scale fatigue; HAQ, health assessment questionnaire; RAQoL, rheumatoid arthritis quality of life questionnaire; WHOQOL-BREF, World Health Organization Quality of Life Questionnaire Short Form; SWEMWBS, Short Warwick Edinburgh Mental Wellbeing Scale

The questionnaire was completed between April-June 2020 (coinciding with UK shielding guidance: March-July 2020) and included eight fields (Fig. 1: (1) demographic questions; (2) COVID-19 related questions; (3) filter question on RA diagnosis—yes/no; (4) PA questions (e.g. PA participation before and during the lockdown, PA barriers and types of exercise performed) and the International Physical Activity Questionnaire—short form (IPAQ) [15]; (5a) those who responded yes to RA diagnosis, answered questions relating to disease characteristics (i.e. Visual analogue scale (VAS) pain, VAS fatigue, Health Assessment Questionnaire (HAQ) and RA quality of life questionnaire (RAQoL)) [16]; (5b) those who responded no to RA diagnosis, completed VAS fatigue and the World Health Organization Quality of Life Questionnaire Short Form (WHOQOL-BREF) [17]; (6) Short Warwick Edinburgh Mental Wellbeing Scale (SWEMWBS) [18]; (7) dietary questions relating to food consumption; (8) participants could add any general comments on how the lockdown affected them. Participants could use the back button to review and change their answers; however, to prevent participants submitting multiple entries, once they finished the survey and submitted their responses, participants could no longer access the survey. Standard guidelines for survey-based research were used in the survey planning, design, validation, dissemination and analysis [19, 20].

### Data management and analysis

Qualtrics automatically creates a Microsoft Excel® spreadsheet with the answers, which was stored on a password protected file. Subsequently all questionnaires were audited prior to analyses in order to invalidate any incorrect or incomplete data. Statistical analyses were conducted using SPSS V.26.0 by the lead investigator (C.B.). Variables were checked for homogeneity of variance, outliers, and normality prior to performing statistical tests. Differences in PA, sedentary behaviour, mental wellbeing, and QoL between RA and non-RA participants were evaluated using either independent samples *t*-test or Mann–Whitney test depending on normality testing. Chi-squared (χ^2^) tests were performed to compare PA changes, PA barriers, body weight, food intake and mental wellbeing categories (as described below) in RA and non-RA participants. Types of exercise performed were examined using a waterfall chart constructed on Microsoft Excel®. The relationship between PA participation and mental wellbeing was examined using Pearson’s correlation coefficient. Data presented as means ± standard deviation (SD) unless otherwise stated and the level of significance was set at *p* < 0.05.

SWEMWBS raw scores were transformed using a conversion table (see Online Resource 2) to facilitate the use of parametric statistical analyses [21]. Three-category versions of mental wellbeing scores were derived: low, medium, and high [18] to allow for analysis using a categorical approach.

RA and non-RA participants completed different QoL questionnaires. RAQoL produces a score of 0–30, with zero indicating high QoL and 30 low. For WHOQOL-BREF four mean domain scores and 2 separate scores concerning overall QoL and general health were calculated; all scores were then transformed to a 0–100 scale using a standardized formula [22], with 0 indicating low QoL and 100 high. There are no recommended categories for either measure.

## Results

### Full sample analysis

All 128 questionnaires were completed in full and only 2 participants confirmed a positive diagnosis for COVID-19. Participants were split into two groups based on RA diagnosis: RA (*n* = 27), non-RA (*n* = 101) (Table 1). There were statistically significant differences between groups for gender, shielding status, age, and height (all *p* < 0.05).

**Table 1:** Baseline characteristics for participants. Anthropometric data presented as mean ± SD

	RA participants (*n* = 27)	Non-RA participants (*n* = 101)	*P* value
*Demographic*			
Male *n* (%)	6 (22)	62 (61)	< 0.001
Female *n* (%)	21 (78)	39 (39)	< 0.001
Shielding *n* (%)	19 (70)	12 (12)	< 0.001
Age (years)	51 ± 13	38 ± 16	< 0.001
*Anthropometric*			
Height (cm)	166.2 ± 12.7	173.5 ± 8.5	0.005
Weight (kg)	78.5 ± 19.1	79.8 ± 16.7	0.489
BMI (kg/m^2^)	28.7 ± 6.2	26.6 ± 5	0.177
*RA disease duration*			
˂ 2 years (%)	10 (37)	–	–
2–5 years (%)	5 (19)	–	–
˃ 5 years (%)	12 (44)	–	–
*Rheumatological treatment*			
Methotrexate (%)	16 (59)	–	–
Sulfasalazine (%)	7 (26)	–	–
Hydroxychloroquine (%)	8 (30)	–	–
Steroids (%)	4 (15)	–	–
Biologics (%)	9 (33)	–	–
NSAIDS (%)	6 (22)	–	–
Paracetamol/Co-codamol (%)	7 (26)	–	–
Other (%)	5 (19)	–	–
No medication	1 (4)	–	–
*Diagnosed medical conditions*			
Cancer (%)	–	2 (2)	0.461
Diabetes (%)	2 (7)	2 (2)	0.15
Heart disease (%)	2 (7)	2 (2)	0.15
Hypertension (%)	6 (22)	10 (10)	0.086
Lung disease (e.g. asthma) (%)	3 (11)	9 (9)	0.728
Mental health issues (%)	7 (26)	11 (11)	0.046
Other (%)	12 (44)	16 (16)	0.001
No other medical conditions	9 (33)	65 (64)	0.004

An overview of the study results for all participants is presented in Table 2. Before the lockdown 48% of RA participants suggested they were active versus 64% of non-RA participants (χ^2^(3) = 5.573, *p* = 0.134). During lockdown 38% of all our participants reported a reduction in their PA participation; among those with RA, reduced PA was more prevalent than among those without RA (59% vs 33%; χ^2^(4) = 10.181, *p* = 0.037) (Table 3). Consequently, self-reported PA (MET minutes/week) during lockdown for RA participants (median: 1160, 95% confidence interval (95% CI): 297, 1848) was significantly lower than non-RA participants (median: 2940, 95% CI: 2376, 3546, *p* < 0.001). However, there was no significant difference in self-reported PA between shielding and not shielding participants, in either RA or non-RA groups (all *p* > 0.05). Also, self-reported sedentary behaviour during lockdown was similar in RA participants and non-RA participants (543.2 ± 213.6 vs 485.5 ± 218 min/day, *p* = 0.230).

**Table 2:** IPAQ, SWEMWBS, RAQoL and WHOQOL-BREF data

	RA (*n* = 27)			Non-RA (*n* = 101)			*p* value
	Shielding (*n* = 19)	Not shielding (*n* = 8)	Total (*n* = 27)	Shielding (*n* = 12)	Not shielding (*n* = 89)	Total (*n* = 101)	
IPAQ (MET minute/week)	1160 (297, 2274)	1089 (0, 2376)	1160 (297, 1848)	3696 (396, 6217)	2932.5 (2346, 3546)	2940 (2376, 3546)	< 0.001*
SWEMWBS (7–35)	19.5 ± 3.5	24 ± 4.2	20.8 ± 4.2	21.3 ± 3.7	22.3 ± 3.4	22.2 ± 3.4	0.080*
WHOQOL-BREF Domain 1 Physical Health (0–100)	–	–	–	59.5 ± 25.5	77.4 ± 13.2	75.3 ± 16.1	–
WHOQOL-BREF Domain 2 Psychological (0–100)	–	–	–	59 ± 22	64.5 ± 15.9	63.8 ± 16.7	–
WHOQOL-BREF Domain 3 Social Relationships (0–100)	–	–	–	62.5 ± 25.5	65.0 ± 19.6	64.7 ± 20.2	–
WHOQOL-BREF Domain 4 Environment (0–100)	–	–	–	70.0 ± 14.2	75.2 ± 13.3	74.6 ± 13.5	–
WHOQOL-BREF Overall QOL (0–100)	–	–	–	66.7 ± 19.5	78.4 ± 16.5	77.0 ± 17.2	–
WHOQOL-BREF General Health (0–100)	–	–	–	45.8 ± 27.9	66.0 ± 26.2	63.6 ± 27.0	–
RAQoL (0–30)	15.9 ± 7.5	3.9 ± 3.0	12.3 ± 8.5	–	–	–	–

All data are mean ± SD except IPAQ (median (95% CI))

^*^Statistically significant difference between RA and non-RA participants

**Table 3:** Changes in PA participation during lockdown and % of RA and non-RA participants

PA change	RA (*n* = 27)	Non-RA (*n* = 101)
More physically active (%)	30	37
They haven’t changed	11	30
Less physically active	59	33

**Fig. 2:**
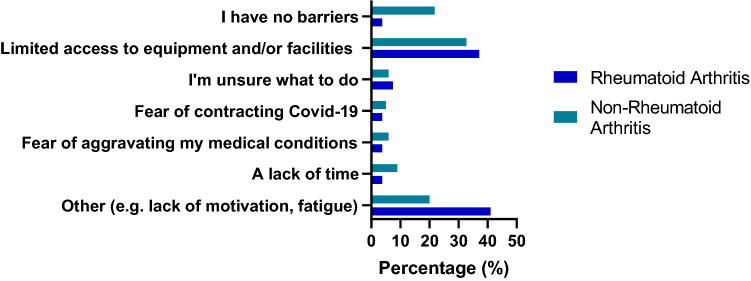
Main barriers to PA in RA and non-RA participants during lockdown. Participants were asked to select their biggest barrier to PA during lockdown. In RA participants, 41% selected Other (e.g. lack of motivation, fatigue) as their biggest barrier, while 37% selected Limited access to equipment and/or facilities as their biggest barrier (versus 33% of non-RA participants). Only 4% of RA participants reported having no barriers to PA during lockdown versus 22% of non-RA participants

**Fig. 3:**
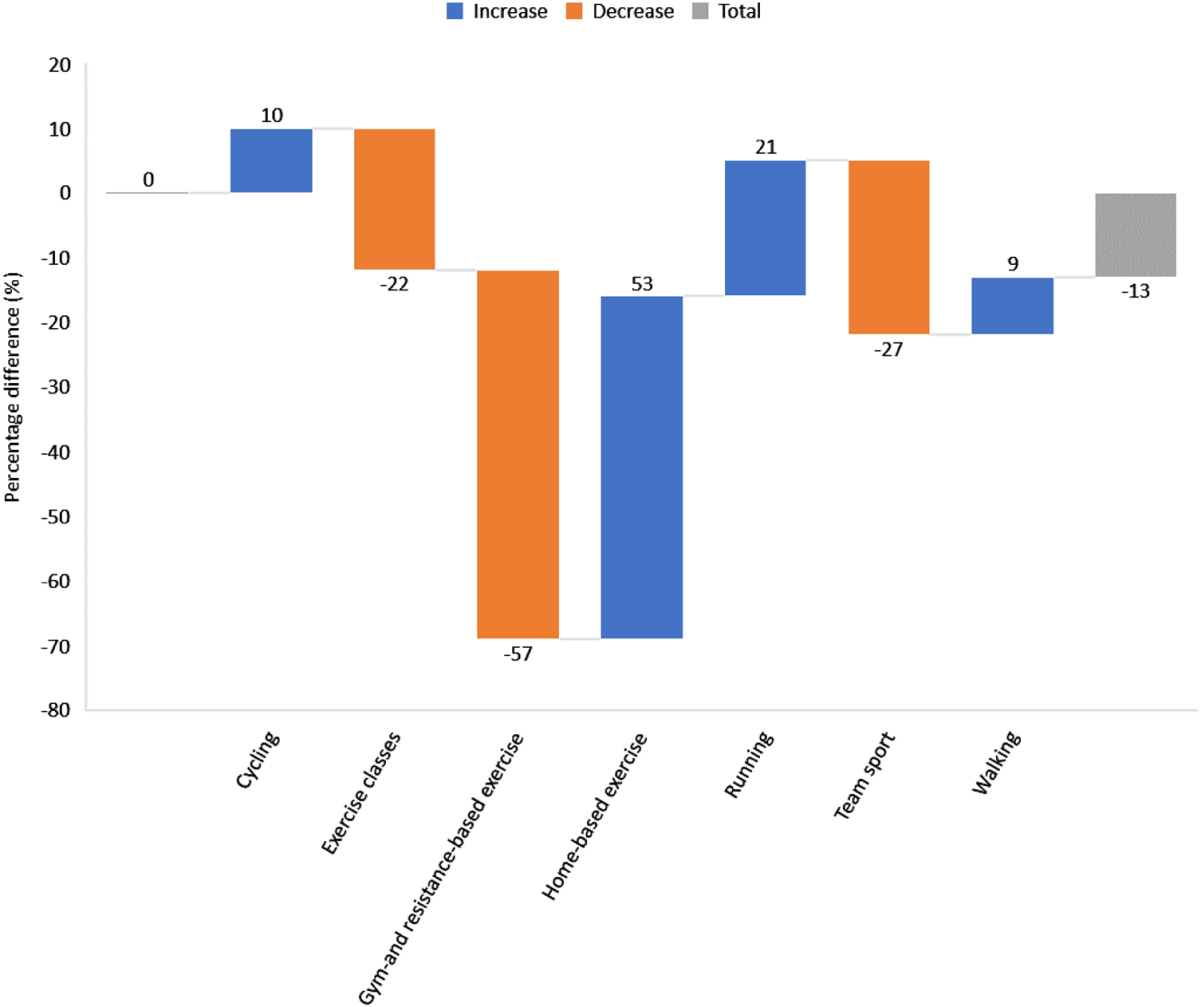
Difference (%) in types of exercise before and during lockdown for all participants. A waterfall chart was constructed to examine types of exercise performed by all participants. Following lockdown orders participation in gym- and resistance-based exercise, team sports and exercise classes all decreased ( – 57%,  – 27% and  – 22% respectively), while participation in home-based exercise, running, cycling and walking increased in all participants (53%, 21%, 10% and 9% respectively)

Limited access to equipment and/or facilities was reported by 37% of people with RA as the biggest PA barrier during lockdown and 33% of those without (χ^2^(6) = 8.931, *p* = 0.177), whilst 4% of RA participants suggested they had no barriers versus 22% of non-RA participants (Fig. 2). Types of exercise performed before lockdown (e.g. exercise classes, gym- and resistance-based exercise and team sports) all stopped during, whilst home-based exercise, running cycling and walking increased in all participants (53%, 21%, 10% and 9%, respectively) (Fig. 3).

Increased body weight was reported by 40% of our participants. People with RA were more likely to report increased body weight vs those without (59% vs 35%; χ^2^(3) = 5.677, *p* = 0.128) (Online Resource 3). In contrast, more non-RA participants indicated eating more during the lockdown than RA participants (25% vs 15%; χ^2^(2) = 1.330, *p* = 0.514). None of these differences were statistically significant.

Mental wellbeing during lockdown was similar among RA participants (20.8 ± 4.2) and non-RA participants (22.2 ± 3.4, 0–35; *p* = 0.080). Furthermore, our results indicated 44% of RA participants and 28% of non-RA participants were classified with low mental wellbeing (χ^2^(2) = 2.832 *p* = 0.243) (Online Resource 4). However, only a weak correlation was identified between PA participation and mental wellbeing among all participants (*r* = 0.225, *p* = 0.011). Also, there was a significant difference in QoL between shielding RA and non-shielding RA participants, where a lower score indicates a higher QoL (15.9 ± 7.5 vs 3.9 ± 3.0, *t*(25) = -5.916, *p* < 0.001).

### Matched group analysis

Due to the substantial group differences in sample size, as well as baseline characteristics, we pursued further analyses where our 27 RA participants were matched with 27 non-RA participants for gender, age, height, weight and BMI (Table 4).

**Table 4:** Baseline characteristics for matched RA and non-RA participants. Anthropometric data presented as mean ± SD

	RA participants (*n* = 27)	Non-RA participants (*n* = 27)	*P* value
*Demographic*			
Male *n* (%)	6 (22)	6 (22)	–
Female *n* (%)	21 (78)	21 (78)	–
Age (years)	51 ± 13	48 ± 18	0.609
*Anthropometric*			
Height (cm)	166.2 ± 12.7	168 ± 9.1	0.938
Weight (kg)	78.5 ± 19.1	79.3 ± 18.6	0.813
BMI (kg/m^2^)	28.7 ± 6.2	28.6 ± 6.1	0.976

Matched group comparisons identified similar trends to full sample analyses, even though some were of lesser significance. Compared to matched non-RA participants, fewer people with RA were physically active before lockdown (67% vs 48%; χ^2^(3) = 6.017, *p* = 0.111), while more people with RA reported further reductions in PA during lockdown (59% vs 41%; χ^2^(2) = 3.199, *p* = 0.202). IPAQ scores remained significantly lower for the RA group vs the matched non-RA group (*p* = 0.001), whilst increased body weight was reported to a similar extent in both groups (RA: 59%; non-RA: 52%). Furthermore, 15% of both RA and matched non-RA participants indicated eating more during the lockdown. However, more RA participants were classified with low mental wellbeing than matched non-RA participants (44% vs 33%; χ^2^(2) = 0.771, *p* = 0.680).

## Discussion

The aim of this study was to evaluate the impact of lockdown on PA participation and any barriers to it, type of exercise performed, body weight changes, mental wellbeing and QoL in people with RA versus people without RA. Among our participants, people with RA were more likely to report reduced PA and increased body weight as a result of lockdown versus those without RA; shielding status did not seem to affect this. However, we have identified similarities in the PA barriers reported by RA and non-RA participants, with limited access to equipment and facilities being a key reason for reduced PA participation. Consequently, exercise classes, gym- and resistance-based exercise and team sports decreased, as consistent with lockdown orders whilst home-based exercise and individual outdoor-based exercise increased in all participants. We also identified a similar percentage of RA and non-RA participants who reported increased food intake during lockdown. Furthermore, more RA participants were classified with low mental wellbeing versus non-RA participants.

Participation rates in PA are significantly lower in people with RA versus the general population [23]. Our findings further support this notion as prior to lockdown, our participants with RA reported lower PA participation versus those without. Unfortunately, the lockdown appears to have further affected PA participation among people with RA, as almost twice as many (59%) reported reduced participation in PA versus non-RA participants (33%). Interestingly, another study found that during lockdown 50% of people with RA significantly decreased the amount of PA they did, indeed the proportion of RA participants who engaged in active mobility (e.g. walking, biking etc.) during lockdown plummeted from 74 to 42% [24]. Also to maintain PA, most participants reported using a digital method (online exercise videos, computer applications etc.) [24]; which we did not examine in this present study. Nevertheless, both studies highlight during lockdown PA decreased in people with RA, which may have implications for disease outcomes.

Decreased PA levels during lockdown have already been reported [9, 25]; with considerations for the impact of shielding [25]. Although in our study PA participation between shielding and non-shielding RA participants was similar. Commonly, people with RA experience more barriers to PA than the general population [4]. Indeed, among our participants with RA, most reported having some barriers to PA, while almost a quarter of people without RA reported no barriers, even during lockdown. Access to facilities and equipment appears to be a major barrier for both groups while other common barriers such as time availability, uncertainty on what to do etc. were not mentioned as much in our study. Among the RA participants “Other (including lack of motivation and fatigue)” was also frequently mentioned. Fatigue is a major feature of RA and associated with joint pain, which may mediate the worsening of disease activity [26]. Exercise may counteract fatigue, but a lack of movement may enhance it, which may lead to people with RA beginning a vicious circle of inactivity, fatigue, and inactivity. Therefore, it is important that PA promotion addresses this, and they start to move.

As expected, due to the measures in place, participation in exercise classes, gym- and resistance-based exercise, and team sports was reduced, while home-based exercise and individual outdoor PA (e.g. cycling, running, and walking) increased during lockdown. Importantly, we identified only a few participants who did no exercise and despite alluding to a change in type of exercise performed, participants who were active before lockdown generally continued doing PA during lockdown. However, whilst most participants remained active, their overall PA participation decreased during lockdown, particularly in RA participants. Therefore, our results have significant implications for people with RA as reduced habitual PA has been shown to exacerbate symptoms and accelerate disease progression [27] Furthermore, daily exercise may in fact help combat certain infections, including COVID-19 by counteracting some of the comorbidities that are more susceptible to severe illness [28], whilst also strengthening the immune system [29].

Moreover, low levels of PA associate with increased body weight in RA [11]. In the present study, more RA participants reported an increase in body weight during lockdown versus non-RA participants (59% and 35% respectively), whilst changes in food intake were similar between participants. Therefore, movement rather than nutritional limitations could account for body weight changes. Obesity in RA may increase the risk for CVD and predispose to higher disease activity [11], but also obese individuals are more likely to develop severe COVID-19 symptoms [30].

Mental wellbeing scores were low in all participants (RA: 20.8 ± 4.2; non-RA: 22.2 ± 3.4), with scores of 18–20 suggesting possible depression [31]. Furthermore, shielding RA participants had the lowest mental wellbeing score (19.5 ± 3.5); whilst there was also a significant difference in QoL between shielding RA and non-shielding RA participants (15.9 ± 7.5 vs 3.9 ± 3.0), where a lower score indicates a better QoL. Recently it was reported the more active their RA was during lockdown (i.e. high disease activity), the greater the impact was on QoL indicators [24], although this association was not explored in the present study so we cannot confirm this finding. Our results may be a consequence of the significant restrictions imposed on shielding RA participants. Previous research has suggested the outdoor environment can induce mental stimulation [32] and therefore improve mental wellbeing. However, we are unable to confirm if the lockdown caused changes in mental wellbeing and QoL, as participants did not complete a pre-lockdown evaluation. A recent study identified PA was positively associated with mental health and psychological wellbeing in RA during COVID-19 [33], which suggests that PA can be imperative for maintaining mental functioning. There are a number of differences with this present study, including different mental health assessment tools. Additionally, we didn’t observe an association between PA levels and mental wellbeing among our participants. Nevertheless, both studies emphasise the importance of encouraging PA for people with RA during the pandemic for mental health and wellbeing.

Our study has several limitations, first due to the rapid onset of the COVID-19 pandemic a convenience sampling approach was adopted and therefore, our results may have been affected by this. For example, we used social media platforms to disseminate our survey; but such platforms do not allow calculation of the denominator of the target population [20] and therefore we were unable to gather an accurate response rate. Furthermore, the cross-sectional study design does not allow for the assessment of causality. There was a small sample size recruited, particularly in the RA group (*n* = 27), which may have underpowered our analysis. There are also several inter-group differences such as number of participants in RA vs non-RA group and demographic differences (e.g. age and % of females in each group). However, we analyzed our data for matched groups and the findings were similar to those of the entire sample. The data were entirely self-reported and therefore, may be subject to bias; for example self-reported PA data is not considered as accurate in comparison to objective assessments (i.e. accelerometers) [34], with participants possibly overestimating PA participation. Collectively these limitations may impact the robustness of our results. Nevertheless, the data was checked for outliers, while the IPAQ is considered a valid measurement tool of PA [15, 35]. This study has provided a novel insight in to how the COVID-19 pandemic has affected people with RA, our findings are still valid and important implications can be made from them.

A number of efficacious vaccines against COVID-19 have been developed and a global vaccination programme is now being undertaken, which could end the pandemic. However, this may be followed by a physical deconditioning pandemic, whereby months of reduced activity caused by the lockdown will impact on all four aspects of physical fitness: strength, stamina, suppleness, and skills. Additionally, there may be deconditioning of the established behavioural routines that supported regular PA (pre-COVID-19) in people with RA. The present study has demonstrated the prolonged period of lockdown has reduced PA participation for people with RA and it is also likely to have further affected their body weight. Therefore, it is important that as lockdown restrictions are lifted, people with RA are targeted specifically with exercise programmes, which aim to increase fitness, but also improve mobility and body composition.

In conclusion, during lockdown, people with RA reduced their already low levels of PA more than those without RA. Also, more RA participants reported an increase in body weight versus their non-RA counterparts. The key reason for reduced PA participation appears to be limited access to equipment and facilities, which has then impacted on types of exercise performed. Therefore, our findings show the lockdown has perhaps unintentionally created restrictions on incidental and volitional PA. The combination of low PA levels and obesity could negatively impact on disease activity and overall health for people with RA. Following the COVID-19 pandemic, PA promotion specifically for people with RA will be needed to prevent a pandemic of inactivity.

## Supplementary Information

Below is the link to the electronic supplementary material.

Supplementary file1 (PDF 639 KB)

Supplementary file2 (PDF 169 KB)

Supplementary file3 (PDF 14 KB)

Supplementary file4 (PDF 13 KB)

## Data Availability

The data underlying this article will be shared on reasonable request to the corresponding author and following ethical or other needed approval.
