# Supplementary material for: Participation in physical activity decreased more in people with rheumatoid arthritis than the general population during the COVID-19 lockdown: a cross-sectional study
Source: Rheumatol Int. 2021 Nov 30;42(2):241–50. doi: 10.1007/s00296-021-05054-4 (PMC8631264; doi:10.1007/s00296-021-05054-4)
Supplement: Supplementary file 1 — Supplementary file1 (PDF 639 KB) [file 296_2021_5054_MOESM1_ESM.pdf]

Title: Participation in physical activity decreased more in people with Rheumatoid Arthritis during the COVID-19 lockdown: a cross-sectional study

Journal: Rheumatology International

Authors: Christopher. Balchin[1], Ai Lyn. Tan[2, 3], Oliver. Wilson[1], Jim. McKenna[1], Antonios. Stavropoulos-Kalinoglou[1]

[1]Leeds Beckett University, Carnegie School of Sport, Leeds, UK

[2] University of Leeds, Leeds Institute of Rheumatic and Musculoskeletal Medicine, Chapel Allerton Hospital, Leeds, UK

[3] Leeds Teaching Hospitals NHS Trust, NIHR Leeds Biomedical Research Centre, Leeds, UK

Corresponding author: Dr Antonios Stavropoulos-Kalinoglou, Leeds Beckett University, Carnegie School of Sport, 225 Fairfax Hall, Headingley Campus, Churchwood Avenue, Leeds, LS6 3QS, A.Stavropoulos@leedsbeckett.ac.uk

Online Resource 1: The self-administered online survey (Qualtrics XM, United States)

CONSENT

Participant Information

What is the purpose of this study?

Social isolation imposed due to the ongoing Covid-19 pandemic has had a major impact on everyday life. We want to better understand the impact of social isolation measures on your daily activities and develop suitable ways to help you through this period.

What will happen if I take part?

You will complete one survey now (lasting 20-25 minutes) and then we will send you three shorter surveys to complete (each lasting 10-15 minutes) during lockdown and in the first month after lockdown. In total you will complete four surveys. You are free to withdraw at any point and without giving a reason.

What will be asked?

We will ask you generic questions about yourself, your overall health and on any conditions you may have been diagnosed with. Then we will ask about your physical activity levels, quality of life, mental well-being and dietary habits.

We will also ask you for an email address so that we can contact you for the follow-up surveys.

Study contacts

If you have further questions regarding this study or want to raise any complaints, please don't hesitate to contact one of the following:

Lead researcher: Christopher Balchin
Telephone: 0113 81 22487
Email: C.Balchin@leedsbeckett.ac.uk
Project Supervisor: Dr Antonios Stavropoulos-Kalinoglou
Telephone: 0113 81 24298
Email: A.Stavropoulos@leedsbeckett.ac.uk

Please clink on the link to read the full Participant Information Sheet

---

Page Break

CON
Consent
I understand that:

My participation is entirely voluntary. I will need to provide an email address so that I can be sent future surveys. Importantly, this email will not be passed on to any third parties and will be removed from my answers before analysis takes place so all information I provide will be anonymised. I am free to withdraw from the study at any point, but due to anonymisation it will not be possible to withdraw my answers after they have been submitted. The data gathered from this study will be stored securely and it will not be possible to identify me in any results shared from this research.

CON-confirm By selecting the box below, you are confirming you are at least 18 years old, you have read and understood the information about this study and you voluntarily agree to take part in this study.

○ Confirm and consent (1)

○ I do not consent (2)

Skip To: End of Survey If By selecting the box below, you are confirming you are at least 18 years old, you have read and u... = I do not consent

---

Page Break

End of Block: Consent

Start of Block: Demographics

DEM **Your Background**
Firstly, we're going to ask some questions about you. This section should take you no more than 5 minutes.

If you want to return to the previous page, scroll to the bottom of the page and click on the back button.
You don't have to complete all of the survey at once, your responses will be saved and you can return to it later.

Country In which country do you currently reside?

▼ Afghanistan (1) ... Zimbabwe (1357)

DoB What is your year of birth?

____

Sex What is your gender?

- Male (1)
- Female (2)
- Other/prefer to not say (3)

Height-1 Do you know your height? Please select one of the options.

- Yes - in feet (1)
- Yes - in centimetres (2)
- No (3)

Display This Question:

If Do you know your height? Please select one of the options. = Yes - in feet

Height-feet What is your height?
Inches (1)
Feet (2)

▼ 4 (1) ... 7 ~ 11 (52)

Display This Question:

If Do you know your height? Please select one of the options. = Yes - in centimetres

Height-cm What is your height?
Centimetres (1)

▼ 130 (1) ... 230 (101)

Weight-1 Do you know your weight?

- ◯ Yes - in stone (1)
- ◯ Yes - in kilograms (2)
- ◯ No (3)

Display This Question:

If Do you know your weight? = Yes - in stone

Weight-stone What is your weight?
Stone (3)
Pounds (4)

▼ 6 (1) ... 26 ~ 13 (315)

Display This Question:

If Do you know your weight? = Yes - in kilograms

Weight-kg What is your weight?
Kilograms (1)

▼ 40 (1) ... 160 (121)

Weight-change Do you feel like your weight has changed since social isolation measures have been introduced?

- ◯ Yes - it has increased a lot (>3kg or >6lb)) (1)
- ◯ Yes - it has increased a little (0-3kg or 0-6lb) (2)
- ◯ No - it is about the same (3)
- ◯ Yes - it has decreased a little (0-3kg or 0-6lb) (4)
- ◯ Yes - it has decreased a lot (>3kg or >6lb) (5)
- ◯ Not sure (6)

Smoking Do you smoke or have you ever smoked?

- ◯ Yes - current smoker (20)
- ◯ Yes - ex smoker (21)
- ◯ Never smoked (23)

Display This Question:

If Do you smoke or have you ever smoked? = Yes - current smoker

Smoking-amount How many cigarettes per day did you smoke on average before the lockdown?

- ◯ 1-5 (1)
- ◯ 6-10 (2)
- ◯ 11-20 (3)
- ◯ More than 20 (4)

Display This Question:

If Do you smoke or have you ever smoked? = Yes - current smoker

Smoking habits Have your smoking habits changed since the lockdown started?

○ I'm smoking much less (1)

○ I'm smoking a bit less (2)

○ It hasn't changed (3)

○ I'm smoking a bit more (4)

○ I'm smoking much more (5)

End of Block: Demographics

Start of Block: Covid-19 status

COV-test
Covid-19 Status

This section should take you no more than 5 minutes.

Have you had a test for Covid-19?

○ Yes (1)

○ No (2)

Display This Question:

If Covid-19 Status This section should take you no more than 5 minutes. Have you had a test for Cov... = Yes

COV-diagnosis Did you test positive for Covid-19?

○ Yes (1)

○ No (2)

○ Waiting for the results (3)

Display This Question:

If Covid-19 Status This section should take you no more than 5 minutes. Have you had a test for Cov... = No

COV-self-diagnosis Do you think you have already had Covid-19, but were not tested?

- ○ Yes (1)
- ○ No (2)
- ○ I'm unsure (3)

COV-SYMP:Y/N Have you experienced any Covid-19 symptoms?

- ○ Yes (1)
- ○ No (2)
- ○ I'm unsure (3)

Display This Question:

If Have you experienced any Covid-19 symptoms? = Yes

COV-SYMP Which symptoms of Covid-19 have you experienced? *Select all that apply.*

- ☐ Aches and pains (1)
- ☐ Dry Cough (2)
- ☐ Fever (3)
- ☐ Loss of smell and/or taste (4)
- ☐ Shortness of breath (5)
- ☐ Sore throat (6)
- ☐ Tiredness (7)
- ☐ Other (please state): (8) ____

COV-SI What is your CURRENT isolation status? *Select all that apply.*

- ☐ I'm self-isolating due to a diagnosis of Covid-19 or possible symptoms (1)
- ☐ I'm self-isolating because I'm categorised as higher risk (2)
- ☐ I'm self-isolating because I live with someone who has been diagnosed with Covid-19 or has possible symptoms (3)
- ☐ I'm not self-isolating but I'm working from home and social distancing (4)
- ☐ I'm not self-isolating and continue to work outside the house (5)
- ☐ Other (please state): (6) ____

End of Block: Covid-19 status

Start of Block: RA diagnosis

RA-diagnosis
Rheumatoid Arthritis Diagnosis

This section should take you no more than 2 minutes.
Have you been diagnosed with Rheumatoid Arthritis?

- ○ Yes (1)
- ○ No (2)

Skip To: End of Block If Rheumatoid Arthritis Diagnosis This section should take you no more than 2 minutes. Have you b... = No

---

Diagnosis duration When was your diagnosis?

- ○ Less than 2 years ago (1)
- ○ Between 2-5 years ago (2)
- ○ More than 5 years ago (3)

RA meds What medication are you taking for your Rheumatoid Arthritis?

- ▢ Biologic agents (e.g. ethanercept, infliximab, rituximab) (1)
- ▢ Folic acid (2)
- ▢ Hydroxychloroquine (3)
- ▢ Leflunomide (4)
- ▢ Methotrexate (5)
- ▢ NSAIDs (e.g. ibuprofen) (6)
- ▢ Paracetamol (7)
- ▢ Steroids (e.g. prednisolone) (8)
- ▢ Sulfasalazine (9)
- ▢ Other (please state): (10) ____
- ▢ I'm not taking any medication (11)

RA meds change Has your medication changed due to the Covid pandemic?

- ○ Yes (1)
- ○ No (2)

Display This Question:

If Has your medication changed due to the Covid pandemic? = Yes

RA meds change-2 Please explain what has changed?

____

____

____

____

____

End of Block: RA diagnosis

Start of Block: PA

PA-1
Physical Activity
This section should take you no more than 10 minutes.

BEFORE social isolation measures were applied how active would you say you were?

- ○ I was very active, exercising more than 5 days a week (1)
- ○ I was active, exercising 3-5 days a week (2)
- ○ I wasn't very active, exercising less than 3 days a week (3)
- ○ I wasn't exercising at all (4)

Skip To: PA-3 If Physical Activity This section should take you no more than 10 minutes. BEFORE social isolation... = I wasn't exercising at all

PA-2 What type of exercise were you doing BEFORE social isolation measures were introduced? *Please select all that apply.*

- ▢ Cycling (1)
- ▢ Gym based exercise (2)
- ▢ High intensity interval exercise (3)
- ▢ Pilates (4)
- ▢ Running (5)
- ▢ Strength (resistance) exercise (6)
- ▢ Team sports (7)
- ▢ Yoga (8)
- ▢ Zumba (9)
- ▢ Other (please state): (10) ____

PA-3
Have your physical activity levels changed since social isolation measures were introduced?

- ○ I'm a lot more physically active (1)
- ○ I'm more physically active (2)
- ○ They haven't changed (3)
- ○ I'm less physically active (4)
- ○ I'm a lot less physically active (5)

PA-4 What type of exercise are you CURRENTLY doing? *Please select all that apply.*

- ☐ Cycling (1)
- ☐ High intensity interval exercise (2)
- ☐ Home based exercise (3)
- ☐ Pilates (4)
- ☐ Running (5)
- ☐ Strength (resistance) exercise (6)
- ☐ Yoga (7)
- ☐ Zumba (8)
- ☐ Other (please state): (9) ____
- ☐ I'm not doing any exercise (10)

PA-5 What types of exercise would you like to do?

____

____

____

____

____

PA-6 How much exercise do you intend to do in the NEXT 7 DAYS?

- ◯ A lot, exercise on more than 5 days (1)
- ◯ A moderate amount, exercise between 3-5 days (2)
- ◯ A little, exercise on less than 3 days (3)
- ◯ None at all (4)

PA-7 What has been the biggest BARRIER that has stopped you doing exercise since the lockdown started?

- ◯ A lack of time (1)
- ◯ Fear of aggravating my medical conditions (2)
- ◯ Fear of contracting Covid-19 (3)
- ◯ I have Covid-19 symptoms (4)
- ◯ I'm unsure what to do (5)
- ◯ Limited access to equipment and/or facilities (6)
- ◯ Other (please state): (7) ____
- ◯ I have no barriers (8)

Skip To: IPAQ If What has been the biggest BARRIER that has stopped you doing exercise since the lockdown started? = I have no barriers

PA-8 What other BARRIERS have stopped you doing exercise since the lockdown started? *Please select those that apply.*

- ☐ A lack of time (1)
- ☐ Fear of aggravating my medical conditions (2)
- ☐ Fear of contracting Covid-19 (3)
- ☐ I have Covid-19 symptoms (4)
- ☐ I'm unsure what to do (5)
- ☐ Limited access to equipment and/or facilities (6)
- ☐ Other (please state): (7) ____
- ☐ I have no other barriers (8)

Page Break

IPAQ The following questions will ask you about the time you spent being physically active in the LAST 7 DAYS. Please answer each question even if you do not consider yourself to be an active person. Please think about the activities you do at work, as part of your house and garden work, to get from place to place, and in your spare time for recreation or exercise.

Think about all the VIGOROUS activities that you did in the LAST 7 DAYS. VIGOROUS physical activities refer to activities that take hard physical effort and make you breathe much harder than normal. Think only about those physical activities that you did for AT LEAST 10 MINUTES at a time.

IPAQ-Vig/Days During the LAST 7 DAYS, on how many days did you do VIGOROUS physical activities like heavy lifting, digging, aerobics, or fast bicycling?
Days (1)

▼ 0 (1) ... 7 (8)

Skip To: IPAQ-Mod If During the LAST 7 DAYS, on how many days did you do VIGOROUS physical activities like heavy lifting... = 0

IPAQ-Vig/Mins How much time did you usually spend doing VIGOROUS physical activities on just one of those days?
Hours (1)
Minutes (2)

▼ 0 (1) ... 16 ~ 55 (221)

IPAQ-Mod Think about all the MODERATE activities that you did in the LAST 7 DAYS. MODERATE activities refer to activities that take moderate physical effort and make you breathe somewhat harder than normal. Think only about those physical activities that you did for AT LEAST 10 MINUTES at a time.

IPAQ-Mod/Days During the LAST 7 DAYS, on how many days did you do MODERATE physical activities like carrying light loads, bicycling at a regular pace, or doubles tennis? DO NOT include walking.
Days (1)

▼ 0 (1) ... 7 (8)

Skip To: IPAQ-Walk If During the LAST 7 DAYS, on how many days did you do MODERATE physical activities like carrying li... = 0

IPAQ-Mod/Mins How much time did you usually spend doing MODERATE physical activities on just one of those days?
Hours (1)
Minutes (2)

▼ 0 (1) ... 16 ~ 55 (221)

IPAQ-Walk Think about the time you spent WALKING in the LAST 7 DAYS. This includes at work and at home, walking to travel from place to place, and any other walking that you have done solely for recreation, exercise, or leisure.

IPAQ-Walk/Days During the LAST 7 DAYS, on how many days did you WALK for AT LEAST 10 MINUTES at a time?
Days (1)

▼ 0 (1) ... 7 (8)

Skip To: IPAQ-Sed If During the LAST 7 DAYS, on how many days did you WALK for AT LEAST 10 MINUTES at a time? = 0

IPAQ-Walk/Mins How much time did you usually spend WALKING on just one of those days?
Hours (1)
Minutes (2)

▼ 0 (1) ... 16 ~ 55 (221)

IPAQ-Sed The last question for this section is about the time you spent SITTING on weekdays during the LAST 7 DAYS. Include time spent at work, at home, while doing course work and during leisure time. This may include time spent sitting at a desk, reading, or sitting or lying down to watch television.

IPAQ-Sed/Mins During the LAST 7 DAYS, how much time did you spend SITTING on a WEEK DAY?
Hours (1)
Minutes (2)

▼ 0 (1) ... 16 ~ 55 (221)

End of Block: PA

Start of Block: RA block

Display This Question:

If Rheumatoid Arthritis Diagnosis   This section should take you no more than 2 minutes. Have you b... = Yes

DAS28
Disease Activity

We're now going to ask you some questions on your Rheumatoid Arthritis. This section should take you about 10 minutes.

Display This Question:

If Rheumatoid Arthritis Diagnosis   This section should take you no more than 2 minutes. Have you b... = Yes

DAS28-GlobalVAS What has been your overall well-being in THE LAST 7 DAYS? *Please indicate using the sliding scale* (0 = best health state and 100 = worst health state).

	0	100
Overall wellbeing ()		

Display This Question:

If Rheumatoid Arthritis Diagnosis   This section should take you no more than 2 minutes. Have you b... = Yes

VAS-Pain How much arthritis pain have you felt in THE LAST 7 DAYS? *Please indicate using the sliding scale* (0 = no pain and 100 = pain as bad as it could possibly be).

	0	100
Pain ()		

Display This Question:

If Rheumatoid Arthritis Diagnosis   This section should take you no more than 2 minutes. Have you b... = Yes

VAS-Fatigue How tired have you felt in the LAST 7 DAYS? *Please indicate using the sliding scale* (0 = no fatigue and 100 = severe fatigue).

	0	100

Fatigue ()	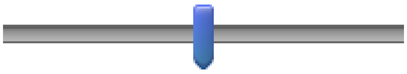

Page Break

Display This Question:

If Rheumatoid Arthritis Diagnosis This section should take you no more than 2 minutes. Have you b... = Yes

HAQ

Health Assessment Questionnaire

Please select the one response which best describes your abilities over the PAST 7 DAYS.

Display This Question:

If Rheumatoid Arthritis Diagnosis This section should take you no more than 2 minutes. Have you b... = Yes

HAQ-DG Dressing and Grooming

ARE YOU ABLE TO:

	Without ANY difficulty (0)	With SOME difficulty (1)	With MUCH difficulty (2)	UNABLE to do (3)
Dress yourself, including tying shoelaces and doing buttons? (1)	○	○	○	○
Shampoo your hair? (2)	○	○	○	○

Display This Question:

If Rheumatoid Arthritis Diagnosis This section should take you no more than 2 minutes. Have you b... = Yes

HAQ-R Rising

ARE YOU ABLE TO:

	Without ANY difficulty (0)	With SOME difficulty (1)	With MUCH difficulty (2)	UNABLE to do (3)
Stand up from an arm-less straight chair? (1)	○	○	○	○
Get in and out of bed? (2)	○	○	○	○

Display This Question:

If Rheumatoid Arthritis Diagnosis   This section should take you no more than 2 minutes. Have you b... = Yes

X→

HAQ-E Eating

ARE YOU ABLE TO:

	Without ANY difficulty (0)	With SOME difficulty (1)	With MUCH difficulty (2)	UNABLE to do (3)
Cut your meat? (1)	○	○	○	○
Lift a full cup or glass to your mouth? (2)	○	○	○	○
Open a new carton of milk (or soap powder)? (3)	○	○	○	○

Display This Question:

If Rheumatoid Arthritis Diagnosis   This section should take you no more than 2 minutes. Have you b... = Yes

X→

HAQ-W Walking

ARE YOU ABLE TO:

	Without ANY difficulty (0)	With SOME difficulty (1)	With MUCH difficulty (2)	UNABLE to do (3)
Walk outdoors on flat ground? (1)	○	○	○	○
Climb up five steps (2)	○	○	○	○

Display This Question:

If Rheumatoid Arthritis Diagnosis   This section should take you no more than 2 minutes. Have you b... = Yes

X→

HAQ-Devices Please select any AIDS or DEVICES that you usually use for any of these activities.

- ☐ Cane (1)
- ☐ Walking frame (2)
- ☐ Built-up or special utensils (3)
- ☐ Crutches (4)
- ☐ Wheelchair (5)
- ☐ Special or built-up chair (6)
- ☐ Devices used for dressing (button hooks, zipper pull, shoe horn) (7)
- ☐ Other (please specify) (8) ____

Display This Question:

If Rheumatoid Arthritis Diagnosis This section should take you no more than 2 minutes. Have you b... = Yes

X→

HAQ-Help Please select any categories for which you usually need help from another person:

- ☐ Dressing and Grooming (1)
- ☐ Eating (2)
- ☐ Rising (3)
- ☐ Walking (4)

Display This Question:

If Rheumatoid Arthritis Diagnosis This section should take you no more than 2 minutes. Have you b... = Yes

X→

HAQ-Hygeine Hygiene

ARE YOU ABLE TO:

	Without ANY difficulty (0)	With SOME difficulty (1)	With MUCH difficulty (2)	UNABLE to do (3)
Wash and dry your entire body? (1)	○	○	○	○
Take a bath? (2)	○	○	○	○
Get on and off the toilet? (3)	○	○	○	○

Display This Question:

If Rheumatoid Arthritis Diagnosis This section should take you no more than 2 minutes. Have you b... = Yes

X→

HAQ-Reach Reach

ARE YOU ABLE TO:

	Without ANY difficulty (0)	With SOME difficulty (1)	With MUCH difficulty (2)	UNABLE to do (3)
Reach and get down a 5lb object (e.g. a bag of potatoes) from just above your head? (1)	○	○	○	○
Bend down to pick up clothing off the floor (2)	○	○	○	○

Display This Question:

If Rheumatoid Arthritis Diagnosis This section should take you no more than 2 minutes. Have you b... = Yes

X→

HAQ-Grip Grip

ARE YOU ABLE TO:

	Without ANY difficulty (0)	With SOME difficulty (1)	With MUCH difficulty (2)	UNABLE to do (3)
Open car doors? (1)	○	○	○	○
Open jars which have been previously opened? (2)	○	○	○	○
Turn taps on and off? (3)	○	○	○	○

Display This Question:

If Rheumatoid Arthritis Diagnosis This section should take you no more than 2 minutes. Have you b... = Yes

HAQ-Activities Activities

ARE YOU ABLE TO:

	Without ANY difficulty (0)	With SOME difficulty (1)	With MUCH difficulty (2)	UNABLE to do (3)
Run errands and shop? (1)	○	○	○	○
Get in and out of a car? (2)	○	○	○	○
Do chores such as vacuuming, housework or light gardening? (3)	○	○	○	○

Display This Question:

If Rheumatoid Arthritis Diagnosis This section should take you no more than 2 minutes. Have you b... = Yes

HAQ-Devices2 Please select any AIDS or DEVICES that you usually use for any of these activities:

- ☐ Raised toilet seat (1)
- ☐ Bath seat (2)
- ☐ Bath rail (3)
- ☐ Long handled appliances for reach (4)
- ☐ Jar opener (for jars previously opened) (5)
- ☐ Other (please specify) (6) ____

Display This Question:

If Rheumatoid Arthritis Diagnosis This section should take you no more than 2 minutes. Have you b... = Yes

X→

HAQ-Help2 Please select any categories for which you need help from another person:

- ☐ Hygiene (1)
- ☐ Gripping and opening things (2)
- ☐ Reach (3)
- ☐ Errands and housework (4)

Page Break

Display This Question:

If Rheumatoid Arthritis Diagnosis This section should take you no more than 2 minutes. Have you b... = Yes

RAQoL

Quality of Life

Please select either YES or NO to the following questions:

Display This Question:

If Rheumatoid Arthritis Diagnosis This section should take you no more than 2 minutes. Have you b... = Yes

RAQoL-1 I have to go to bed earlier than I would like to.

○ Yes (1)

○ No (0)

Display This Question:

If Rheumatoid Arthritis Diagnosis This section should take you no more than 2 minutes. Have you b... = Yes

RAQoL-2 I'm afraid of people touching me.

○ Yes (1)

○ No (0)

Display This Question:

If Rheumatoid Arthritis Diagnosis This section should take you no more than 2 minutes. Have you b... = Yes

RAQoL-3 It's difficult to find comfortable shoes that I like.

○ Yes (1)

○ No (0)

Display This Question:

If Rheumatoid Arthritis Diagnosis This section should take you no more than 2 minutes. Have you b... = Yes

RAQoL-4 I avoid crowds because of my condition.

- Yes (1)
- No (0)

Display This Question:

If Rheumatoid Arthritis Diagnosis This section should take you no more than 2 minutes. Have you b... = Yes

RAQoL-5 I have difficulty dressing.

- Yes (1)
- No (0)

Display This Question:

If Rheumatoid Arthritis Diagnosis This section should take you no more than 2 minutes. Have you b... = Yes

RAQoL-6 I find it difficult to walk to the shops.

- Yes (1)
- No (0)

Display This Question:

If Rheumatoid Arthritis Diagnosis This section should take you no more than 2 minutes. Have you b... = Yes

RAQoL-7
Jobs about the house take me a long time.

Yes (1)

No (0)

Display This Question:

If Rheumatoid Arthritis Diagnosis This section should take you no more than 2 minutes. Have you b... = Yes

RAQoL-8 I sometimes have problems using the toilet.

Yes (1)

No (0)

Display This Question:

If Rheumatoid Arthritis Diagnosis This section should take you no more than 2 minutes. Have you b... = Yes

RAQoL-9 I often get frustrated.

Yes (1)

No (0)

Display This Question:

If Rheumatoid Arthritis Diagnosis This section should take you no more than 2 minutes. Have you b... = Yes

RAQoL-10 I have to keep stopping what I am doing to rest.

Yes (1)

No (0)

Display This Question:

If Rheumatoid Arthritis Diagnosis   This section should take you no more than 2 minutes. Have you b... = Yes

RAQoL-11 I have difficulty using a knife and fork.

- Yes (1)
- No (0)

Display This Question:

If Rheumatoid Arthritis Diagnosis   This section should take you no more than 2 minutes. Have you b... = Yes

RAQoL-12 I find it hard to concentrate.

- Yes (1)
- No (0)

Display This Question:

If Rheumatoid Arthritis Diagnosis   This section should take you no more than 2 minutes. Have you b... = Yes

RAQoL-13 Sometimes I just want to be left alone.

- Yes (1)
- No (0)

Display This Question:

If Rheumatoid Arthritis Diagnosis   This section should take you no more than 2 minutes. Have you b... = Yes

RAQoL-14 I find it difficult to walk very far.

- Yes (1)
- No (0)

Display This Question:

If Rheumatoid Arthritis Diagnosis This section should take you no more than 2 minutes. Have you b... = Yes

RAQoL-15 I try to avoid shaking hands with people.

- Yes (1)
- No (0)

Display This Question:

If Rheumatoid Arthritis Diagnosis This section should take you no more than 2 minutes. Have you b... = Yes

RAQoL-16 I often get depressed.

- Yes (1)
- No (0)

Display This Question:

If Rheumatoid Arthritis Diagnosis This section should take you no more than 2 minutes. Have you b... = Yes

RAQoL-17 I'm unable to join activities with my family or friends.

- Yes (1)
- No (0)

Display This Question:

If Rheumatoid Arthritis Diagnosis This section should take you no more than 2 minutes. Have you b... = Yes

X→

RAQoL-18 I have problems taking a bath/shower.

- ○ Yes (1)
- ○ No (0)

Display This Question:

If Rheumatoid Arthritis Diagnosis This section should take you no more than 2 minutes. Have you b... = Yes

X→

RAQoL-19 I sometimes have a good cry because if my condition.

- ○ Yes (1)
- ○ No (0)

Display This Question:

If Rheumatoid Arthritis Diagnosis This section should take you no more than 2 minutes. Have you b... = Yes

X→

RAQoL-20 My condition limits the places I can go.

- ○ Yes (1)
- ○ No (0)

Display This Question:

If Rheumatoid Arthritis Diagnosis This section should take you no more than 2 minutes. Have you b... = Yes

X→

RAQoL-21 I feel tired whatever I do.

- Yes (1)
- No (0)

Display This Question:

If Rheumatoid Arthritis Diagnosis This section should take you no more than 2 minutes. Have you b... = Yes

RAQoL-22 I feel dependent on others.

- Yes (1)
- No (0)

Display This Question:

If Rheumatoid Arthritis Diagnosis This section should take you no more than 2 minutes. Have you b... = Yes

RAQoL-23 Mycondition is always on my mind.

- Yes (1)
- No (0)

Display This Question:

If Rheumatoid Arthritis Diagnosis This section should take you no more than 2 minutes. Have you b... = Yes

RAQoL-24 I often get angry with myself.

- Yes (1)
- No (0)

Display This Question:

If Rheumatoid Arthritis Diagnosis This section should take you no more than 2 minutes. Have you b... = Yes

RAQoL-25 It's too much effort to go out and see people.

- Yes (1)
- No (0)

Display This Question:

If Rheumatoid Arthritis Diagnosis This section should take you no more than 2 minutes. Have you b... = Yes

RAQoL-26 I sleep badly at night.

- Yes (1)
- No (0)

Display This Question:

If Rheumatoid Arthritis Diagnosis This section should take you no more than 2 minutes. Have you b... = Yes

RAQoL-27 I find it difficult to take care of the people I am close to.

- Yes (1)
- No (0)

Display This Question:

If Rheumatoid Arthritis Diagnosis This section should take you no more than 2 minutes. Have you b... = Yes

RAQoL-28 I feel that I'm unable to control my condition.

◯ Yes (1)

◯ No (0)

Display This Question:

If Rheumatoid Arthritis Diagnosis This section should take you no more than 2 minutes. Have you b... = Yes

RAQoL-29 I avoid physical contact.

◯ Yes (1)

◯ No (0)

Display This Question:

If Rheumatoid Arthritis Diagnosis This section should take you no more than 2 minutes. Have you b... = Yes

RAQoL-30 I'm limited in the clothes I can wear.

◯ Yes (1)

◯ No (0)

Page Break

End of Block: RA block

Start of Block: Other Med Conditions

MC-Diagnosis
Other Medical Conditions
This section should take you no more than 2 minutes.

Do you have any OTHER medical conditions? *Please select all that apply.*

- ▢ Cancer (1)
- ▢ Dementia (2)
- ▢ DIabetes (3)
- ▢ Heart disease (4)
- ▢ High blood pressure (5)
- ▢ Lung disease (e.g. asthma or COPD) (6)
- ▢ Mental health issues (e.g. depression) (7)
- ▢ Stroke (8)
- ▢ Other (please state): (9) ____
- ▢ I have no other medical conditions (10)

MC-Meds Are you taking any medication? This DOES NOT include any Rheumatoid Arthritis medication.

- ▢ Aspirin (1)
- ▢ NSAIDs (e.g. ibuprofen) (2)
- ▢ Blood pressure medication (3)
- ▢ Cholesterol medication (4)
- ▢ Other (please state): (5) ____
- ▢ I'm not taking any medication (6)

Skip To: End of Block If Are you taking any medication? This DOES NOT include any Rheumatoid Arthritis medication. = I'm not taking any medication

MC-Meds change Has your medication changed due to the Covid-19 pandemic?

- Yes (1)
- No (2)

Display This Question:

If Has your medication changed due to the Covid-19 pandemic? = Yes

MC-Meds change 2 Please explain what has changed?

____

____

____

____

____

Page Break

End of Block: Other Med Conditions

Start of Block: Non-RA QoL

Display This Question:

If Rheumatoid Arthritis Diagnosis This section should take you no more than 2 minutes. Have you b... = No

Non-RA-QoL
Quality of Life

The following questions will ask how you feel about your quality of life, health, or other areas of your life. Please answer all the questions. If you are unsure about which response to give to a question, *please choose the one that appears most appropriate.* This can often be your first response. This section should take you about 5 minutes.

Please keep in mind your standards, hopes, pleasures and concerns. We ask that you think about your life in the LAST 2 WEEKS.

Please read each question, assess your feelings, and select the response on the scale for each question that gives the best answer for you.

Display This Question:

If Rheumatoid Arthritis Diagnosis This section should take you no more than 2 minutes. Have you b... = No

NonRAQoL-1 How would you rate your quality of life?

- ◯ Very poor (1)
- ◯ Poor (2)
- ◯ Neither poor nor good (3)
- ◯ Good (4)
- ◯ Very good (5)

Display This Question:

If Rheumatoid Arthritis Diagnosis This section should take you no more than 2 minutes. Have you b... = No

NonRAQoL-2 How satisfied are you with your health?

- Very dissatisfied (1)
- Dissatisfied (2)
- Neither satisfied nor dissatisfied (3)
- Satisfied (4)
- Very satisfied (5)

Page Break

Display This Question:

If Rheumatoid Arthritis Diagnosis This section should take you no more than 2 minutes. Have you b... = No

NonRAQoL:3--9 The following questions ask about how much you have experienced certain things in the LAST 2 WEEKS.

	Not at all (1)	A little (2)	A moderate amount (3)	Very much (4)	Extremely (5)
To what extent do you feel that physical pain prevents you from doing what you need to do? (NonRAQoL-3)	◯	◯	◯	◯	◯
How much do you need any medical treatment to function in your daily life? (NonRAQoL-4)	◯	◯	◯	◯	◯
How much do you enjoy life? (NonRAQoL-5)	◯	◯	◯	◯	◯
To what extent do you feel your life to be meaningful? (NonRAQoL-6)	◯	◯	◯	◯	◯
How well are you able to concentrate? (NonRAQoL-7)	◯	◯	◯	◯	◯
How safe do you feel in your daily life? (NonRAQoL-8)	◯	◯	◯	◯	◯
How healthy is your physical environment? (NonRAQoL-9)	◯	◯	◯	◯	◯

Page Break

Display This Question:

If Rheumatoid Arthritis Diagnosis This section should take you no more than 2 minutes. Have you b... = No

NonRAQoL:10-14 The following questions ask about how completely you experience or were able to do certain things in the LAST 2 WEEKS.

	Not at all (1)	A little (2)	Moderately (3)	Mostly (4)	Completely (5)
Do you have enough energy for everyday life? (NonRAQoL-10)	o	o	o	o	o
Are you able to accept your bodily appearance? (NonRAQoL-11)	o	o	o	o	o
Have you enough money to meet your needs? (NonRAQoL-12)	o	o	o	o	o
How available to you is the information that you need in your day-to-day life? (NonRAQoL-13)	o	o	o	o	o
To what extent do you have the opportunity for leisure activities? (NonRAQoL-14)	o	o	o	o	o

Display This Question:

If Rheumatoid Arthritis Diagnosis This section should take you no more than 2 minutes. Have you b... = No

NonRAQoL:15 The following question asks about how able you were to get around in the LAST 2 WEEKS.

	Very poor (1)	Poor (2)	Neither poor nor good (3)	Good (4)	Very good (5)
How well are you able to get around? (NonRAQoL-15)	○	○	○	○	○

Page Break

Display This Question:

If Rheumatoid Arthritis Diagnosis   This section should take you no more than 2 minutes. Have you b... = No

NonRAQoL:16-25 The following questions ask you to say how good or satisfied you have felt about various aspects of your life over the LAST 2 WEEKS.

	Very dissatisfied (1)	Dissatisfied (2)	Neither satisfied nor dissatisfied (3)	Satisfied (4)	Very satisfied (5)
How satisfied are you with your sleep? (NonRAQoL-16)	○	○	○	○	○
How satisfied are you with your ability to perform your daily living activities? (NonRAQoL-17)	○	○	○	○	○
How satisfied are you with your capacity for work? (NonRAQoL-18)	○	○	○	○	○
How satisfied are you with yourself? (NonRAQoL-19)	○	○	○	○	○
How satisfied are you with your personal relationships? (NonRAQoL-20)	○	○	○	○	○
How satisfied are you with your sex life? (NonRAQoL-21)	○	○	○	○	○
How satisfied are you with the support you get from your friends? (NonRAQoL-22)	○	○	○	○	○
How satisfied are you with the conditions of your living place? (NonRAQoL-23)	○	○	○	○	○

How satisfied are you with your access to health services? (NonRAQoL-24)	○	○	○	○	○
How satisfied are you with your transport? (NonRAQoL-25)	○	○	○	○	○

Page Break

Display This Question:

If Rheumatoid Arthritis Diagnosis This section should take you no more than 2 minutes. Have you b... = No

NonRAQoL:26 The following question refers to how often you have felt or experienced certain things in the LAST 2 WEEKS.

	Never (1)	Seldom (2)	Quite often (3)	Very often (4)	Always (5)
How often do you have negative feelings such as blue mood, despair, anxiety, depression? (NonRAQoL-26)	○	○	○	○	○

Page Break

Display This Question:

If Rheumatoid Arthritis Diagnosis This section should take you no more than 2 minutes. Have you b... = No

NonRA-fatigue How tired have you felt in the LAST 7 DAYS? *Please indicate using the sliding scale* (0 = no fatigue and 100 = severe fatigue).

	0	100
Fatigue ()		

Page Break

End of Block: Non-RA QoL

Start of Block: Mental well-being

SWEMWBS:1-7
Mental Well-being

Below are statements about feelings and thoughts. *Please tick the box* that best describes your experience of each over the LAST 2 WEEKS. This section should take you about 2 minutes.

	None of the time (1)	Rarely (2)	Some of the time (3)	Often (4)	All of the time (5)
I've been feeling optimistic about the future (SWEMWBS-1)	○	○	○	○	○
I've been feeling useful (SWEMWBS-2)	○	○	○	○	○
I've been feeling relaxed (SWEMWBS-3)	○	○	○	○	○
I've been dealing with problems well (SWEMWBS-4)	○	○	○	○	○
I've been thinking clearly (SWEMWBS-5)	○	○	○	○	○
I've been feeling close to other people (SWEMWBS-6)	○	○	○	○	○
I've been able to make up my own mind about things (SWEMWBS-7)	○	○	○	○	○

SWEMWBS-copyright Warwick-Edinburgh Mental Well-being Scale (WEMWBS) copyright University of Warwick 2006, all rights reserved.

End of Block: Mental well-being

Start of Block: Diet

Diet

Diet

You will now be asked a couple of questions about your diet. Please answer as truthfully as possible. This section should take you about 2 minutes.

Since social isolation measures were introduced:

	Agree (1)	No change (2)	Disagree (3)
I'm eating healthier options (Diet_1)	○	○	○
I'm eating more fruit and vegetables (Diet_2)	○	○	○
I'm eating more meat (Diet_3)	○	○	○
I'm eating more dairy products (Diet_4)	○	○	○
I'm eating more confectionery (Diet_5)	○	○	○
I'm drinking more alcohol (Diet_6)	○	○	○

End of Block: Diet

Start of Block: Email

Email What is the email address that you would like us to send the follow-up surveys to? This will not be shared with any third parties.

____

End of Block: Email
