# Supplementary material for: Participation in physical activity decreased more in people with rheumatoid arthritis than the general population during the COVID-19 lockdown: a cross-sectional study
Source: Rheumatol Int. 2021 Nov 30;42(2):241–50. doi: 10.1007/s00296-021-05054-4 (PMC8631264; doi:10.1007/s00296-021-05054-4)
Supplement: Supplementary file 2 — Supplementary file2 (PDF 169 KB) [file 296_2021_5054_MOESM2_ESM.pdf]

Title: Participation in physical activity decreased more in people with Rheumatoid Arthritis during the COVID-19 lockdown: a cross-sectional study

Journal: Rheumatology International

Authors: Christopher. Balchin[1], Ai Lyn. Tan[2, 3], Oliver. Wilson[1], Jim. McKenna[1], Antonios. Stavropoulos-Kalinoglou[1]

[1]Leeds Beckett University, Carnegie School of Sport, Leeds, UK

[2] University of Leeds, Leeds Institute of Rheumatic and Musculoskeletal Medicine, Chapel Allerton Hospital, Leeds, UK

[3] Leeds Teaching Hospitals NHS Trust, NIHR Leeds Biomedical Research Centre, Leeds, UK

Corresponding author: Dr Antonios Stavropoulos-Kalinoglou, Leeds Beckett University, Carnegie School of Sport, 225 Fairfax Hall, Headingley Campus, Churchwood Avenue, Leeds, LS6 3QS, A.Stavropoulos@leedsbeckett.ac.uk

Online Resource 2: Raw score to metric score conversion table for SWEMWBS. Transformed scores were subsequently used for parametric analyses

Raw Score (7-35)	Metric Score (7-35)
7	7.00
8	9.51
9	11.25
10	12.40
11	13.33
12	14.08
13	14.75
14	15.32
15	15.84
16	16.36
17	16.88
18	17.43
19	17.98
20	18.59
21	19.25
22	19.98
23	20.73
24	21.54
25	22.35
26	23.21
27	24.11
28	25.03
29	26.02
30	27.03
31	28.13
32	29.31
33	30.70
34	32.55
35	35.00
