# Supplementary material for: Participation in physical activity decreased more in people with rheumatoid arthritis than the general population during the COVID-19 lockdown: a cross-sectional study
Source: Rheumatol Int. 2021 Nov 30;42(2):241–50. doi: 10.1007/s00296-021-05054-4 (PMC8631264; doi:10.1007/s00296-021-05054-4)
Supplement: Supplementary file 3 — Supplementary file3 (PDF 14 KB) [file 296_2021_5054_MOESM3_ESM.pdf]

Title: Participation in physical activity decreased more in people with Rheumatoid Arthritis during the COVID-19 lockdown: a cross-sectional study

Journal: Rheumatology International

Authors: Christopher. Balchin[1], Ai Lyn. Tan[2, 3], Oliver. Wilson[1], Jim. McKenna[1], Antonios. Stavropoulos-Kalinoglou[1]

[1]Leeds Beckett University, Carnegie School of Sport, Leeds, UK

[2] University of Leeds, Leeds Institute of Rheumatic and Musculoskeletal Medicine, Chapel Allerton Hospital, Leeds, UK

[3] Leeds Teaching Hospitals NHS Trust, NIHR Leeds Biomedical Research Centre, Leeds, UK

Corresponding author: Dr Antonios Stavropoulos-Kalinoglou, Leeds Beckett University, Carnegie School of Sport, 225 Fairfax Hall, Headingley Campus, Churchwood Avenue, Leeds, LS6 3QS, A.Stavropoulos@leedsbeckett.ac.uk

Online Resource 3: Self-reported body weight changes during lockdown and % of RA and non-RA participants. More RA participants reported increased body weight during lockdown than non-RA participants (59% vs 35%)

Body weight change	RA (n = 27)	Non-RA (n = 101)
Increased	59	35
No change	22	36
Decreased	15	26
Not sure	4	3
