# Supplementary material for: Participation in physical activity decreased more in people with rheumatoid arthritis than the general population during the COVID-19 lockdown: a cross-sectional study
Source: Rheumatol Int. 2021 Nov 30;42(2):241–50. doi: 10.1007/s00296-021-05054-4 (PMC8631264; doi:10.1007/s00296-021-05054-4)
Supplement: Supplementary file 4 — Supplementary file4 (PDF 13 KB) [file 296_2021_5054_MOESM4_ESM.pdf]

Title: Participation in physical activity decreased more in people with Rheumatoid Arthritis during the COVID-19 lockdown: a cross-sectional study

Journal: Rheumatology International

Authors: Christopher. Balchin[1], Ai Lyn. Tan[2, 3], Oliver. Wilson[1], Jim. McKenna[1], Antonios. Stavropoulos-Kalinoglou[1]

[1]Leeds Beckett University, Carnegie School of Sport, Leeds, UK

[2] University of Leeds, Leeds Institute of Rheumatic and Musculoskeletal Medicine, Chapel Allerton Hospital, Leeds, UK

[3] Leeds Teaching Hospitals NHS Trust, NIHR Leeds Biomedical Research Centre, Leeds, UK

Corresponding author: Dr Antonios Stavropoulos-Kalinoglou, Leeds Beckett University, Carnegie School of Sport, 225 Fairfax Hall, Headingley Campus, Churchwood Avenue, Leeds, LS6 3QS, A.Stavropoulos@leedsbeckett.ac.uk

Online Resource 4: Mental wellbeing categories and % of RA and non-RA participants. Three-category versions of mental wellbeing were derived from transformed SWEMWBS scores: low, medium and high

Mental wellbeing categories	RA (n = 27)	Non-RA (n = 101)
Low (%)	44	28
Moderate	48	64
High	8	8
